# Genome-Wide Identification and Expression Analyses of the Chitinase Gene Family in Response to White Mold and Drought Stress in Soybean (*Glycine max*)

**DOI:** 10.3390/life12091340

**Published:** 2022-08-29

**Authors:** Peiyun Lv, Chunting Zhang, Ping Xie, Xinyu Yang, Mohamed A. El-Sheikh, Daniel Ingo Hefft, Parvaiz Ahmad, Tuanjie Zhao, Javaid Akhter Bhat

**Affiliations:** 1 National Center for Soybean Improvement, State Key Laboratory of Crop Genetics and Germplasm Enhancement, Nanjing Agricultural University, Nanjing 210095, China; 2 Botany and Microbiology Department, College of Science, King Saud University, Riyadh 11451, Saudi Arabia; 3 School of Chemical Engineering, Edgbaston Campus, University of Birmingham, Birmingham B15 2TT, UK; 4 Department of Botany, GDC, Pulwama 192301, Jammu and Kashmir, India

**Keywords:** *Glycine max* L., PR proteins, chitinase, genome-wide, plant stresses

## Abstract

Chitinases are enzymes catalyzing the hydrolysis of chitin that are present on the cell wall of fungal pathogens. Here, we identified and characterized the chitinase gene family in cultivated soybean (*Glycine max* L.) across the whole genome. A total of 38 chitinase genes were identified in the whole genome of soybean. Phylogenetic analysis of these chitinases classified them into five separate clusters, I–V. From a broader view, the I–V classes of chitinases are basically divided into two mega-groups (X and Y), and these two big groups have evolved independently. In addition, the chitinases were unevenly and randomly distributed in 17 of the total 20 chromosomes of soybean, and the majority of these chitinase genes contained few introns (≤2). Synteny and duplication analysis showed the major role of tandem duplication in the expansion of the chitinase gene family in soybean. Promoter analysis identified multiple cis-regulatory elements involved in the biotic and abiotic stress response in the upstream regions (1.5 kb) of chitinase genes. Furthermore, qRT-PCR analysis showed that pathogenic and drought stress treatment significantly induces the up-regulation of chitinase genes belonging to specific classes at different time intervals, which further verifies their function in the plant stress response. Hence, both in silico and qRT-PCR analysis revealed the important role of the chitinases in multiple plant defense responses. However, there is a need for extensive research efforts to elucidate the detailed function of chitinase in various plant stresses. In conclusion, our investigation is a detailed and systematic report of whole genome characterization of the chitinase family in soybean.

## 1. Introduction

Plants, being immobile, are often subjected to different environmental stresses that lead to a decrease in plant growth and productivity [1,2]. However, to combat these external threats, plants have developed well established defense mechanisms. For example, a small group of heterogeneous proteins called pathogenesis-related (PR) proteins are produced following the attack of disease pathogens, and these proteins plays critical role in inducing plants’ potential to resist pathogen attack [3,4]. Many studies have documented the accumulation and activation of these proteins under multiple abiotic stresses, and thus they are recognized as part of multiple defense systems. Up to now, many families of PR proteins have been characterized [3]; among them, the PR3 family consist of chitinases enzymes that inhibit fungal growth by degrading heterogenous polysaccharide (chitin), a major component of the fungi cell wall [4]. Under normal conditions, these proteins are expressed at basal level; however, pathogen attack or abiotic stress such as drought increases their expression considerably, resulting in systemic acquired resistance (SAR) [4].

Chitinases are ubiquitous in nature and are found in living organisms across different kingdoms of life [5]. The proteins are categorized into two glycosyl hydrolases (GH) families, GH18 & GH19, based on the presence of specific catalytic domains [6]. In addition, by considering the different characteristics of chitinases such as structure, catalytic reaction, phylogenetic relationship and specificity to inhibitors, etc., these chitinases represent five distinct classes (classes I–V) [4]. The members of the GH19 family are specifically found in plants only; however, GH18 family members are widely distributed across different kingdoms, including plants. A lack of chitin in the plant cell wall and other tissue parts makes chitinase an important component of the plant defense system. Chitinase has been documented to the control positive feedback cycle in the plant defense system [7]. This pathway is used by plants in the regulation of plant defense reactions against fungal pathogens [8]. Hence, the chitinases are important targets for enhancing plant growth, especially under environment stresses [9]. To this end, recent studies have also documented the role of chitinase in abiotic situations such as salinity and water deficit conditions [10,11,12].

Soybean (*Glycine max* L. Merr.), an important legume crop, possesses high levels of edible oil and protein in its seed [13]. However, many environment stresses, including both biotic and abiotic conditions, have a negative influence on soybean growth and yield, and the frequency of these stress events has increased due to the changing global climate [14]. Among the biotic stresses, pathogenic diseases such as white mold (caused by *Sclerotinia sclerotiorum*) are a major stress affecting the growth, yield and quality of soybean [15,16]. White mold disease is documented as the fourth major cause of yield losses in soybean [17]. Lack of information about the genes regulating disease resistance is the major hindrance to developing pathogenic-resistant cultivars [18], and the phenotypic evaluation of disease scoring in the field is also technically challenging. Development of resistant cultivars against pathogens requires the identification of underlying genes. The gene family of chitinase has been identified in multiple species, and research studies have confirmed its role against the invasion of fungal pathogens [3,19]; for example, transgenic lines of chitinase genes possess increased resistance to pathogens of fungal origin [3,5]. To this end, chitinases are documented to modulates abiotic stress responses, such as to drought in various plant species [6,11,20]. However, until now, the gene family has not been identified and characterized at the whole genome level in *Glycine max* L. Nevertheless, there are research studies that have used chitinase genes from other organisms to develop transgenic soybean lines [21].

Until now, almost negligible efforts have been made to characterize and identify the chitinase gene family in soybean at the whole genome scale. However, the availability of the whole genome sequence of crop plants is allowing characterization of the whole gene families in plants. In this context, the whole genome sequence of the soybean plant is freely available in public databases (SoyBase and Phytozome); hence, in the current investigation, we identify and characterize the chitinase gene family at the genome-wide scale in soybean. In addition, we also studied the response of the identified chitinase genes under pathogenic attack and drought stress, to confirm their role in plant defense.

## 2. Materials and Methods 

### 2.1. Identifying Chitinase Genes in Soybean

For chitinase gene family identification in soybean, the whole genome sequences of soybean were downloaded from the Phytozome database (https://phytozome-next.jgi.doe.gov/ (accessed on 11 November 2019)), using the *Glycine max Wm82.a2.v1*. This genome sequence was used to develop the protein local database of soybean, using Bioedit ver 7.2 software. Moreover, the 24 known chitinase genes of *Arabidopsis thaliana* freely available at the TAIR database (https://www.arabidopsis.org/ (accessed on 11 November 2019)) were used as a query sequence to identify putative orthologs in soybean, using BLASTp [22]. The e-value <10^−5^ and bit scores >100 were the fitted parameters used to pick out high scoring pairs (HSPs). Redundant hits possessing highest similarity were eliminated to select the unique sequences. To confirm the Glyco_hydro_18 or Glyco_hydro_19 conserved domains, we submitted all identified unique sequences to NCBI-The Conserved Domain Database (https://www.ncbi.nlm.nih.gov/cdd/?term=) (accessed on 17 November 2019).

### 2.2. Phylogenetic Analysis and Multiple Sequence Alignment

Protein sequences of chitinases were aligned using the CLUSTALW function present in MEGA 7.0 [23]. The neighbor-joining method and a bootstrap value of 1000 were used to develop the phylogenetic tree. Chitinases of cultivated soybean (*Glycine max* L.) plus 24 chitinases of *Arabidopsis thaliana* were utilized to develop the phylogenetic tree. Grouping of the chitinases were based on the different chitinase classes (I–V) of *A. thaliana.* Finally, using EvolView (https://www.evolgenius.info//evolview/#login (accessed on 2 December 2019)), the evolutionary trees were developed.

### 2.3. Structure Analysis and Chromosomal Location of Chitinase Genes

The ProtParam database (https://web.expasy.org/protparam/ (accessed on 7 December 2019)), an online program for determining physical protein properties such the molecular weight (MW), length of protein and isoelectric points (pI), was utilized in tge present study for chitinase proteins [24]. The genomic and coding sequence of all chitinases genes were collected from an online database (Phytozome); and gene structures (i.e., exon-intron structures) analysis was performed using the online Gene Structure Display Server tool (http://gsds.gao-lab.org/ (accessed on 7 December 2019)). Chromosomal location information of individual genes of chitinase was obtained from the Phytozome database (https://phytozome-next.jgi.doe.gov/ (accessed on 10 December 2019)); and chromosomal maps were developed with MapChat software (www.https://mapchat.ca/ (accessed on 15 December 2019)).

### 2.4. Promoter Analysis and Three-Dimensional (3D) Structure of Chitinase Genes

The PlantCARE Database (https://bioinformatics.psb.ugent.be/webtools/plantcare/html/ (accessed on 19 December 2019)) was utilized for analysis of cis-regulatory elements in the promotor region (upstream region of 1.5 kb) of chitinase [3].

PHYRE2 server software (http://www.sbg.bio.ic.ac.uk/phyre2/html/page.cgi?id=help (accessed on 19 December 2019)) was used for generating three-dimensional (3D) models, and the thresholds were kept as alignment coverage >65% and confidence = 100%. The transmembrane helix and topology of chitinases proteins were predicted by the MEMSAT-SVM prediction method, available at the PSIPRED online site (https://bio.tools/memsat-svm (accessed on 20 December 2019)).

### 2.5. Synteny and Duplication Analysis

The syntenic information about *Glycine max* and *Arabidopsis thaliana* was downloaded from the Phytozome database (https://phytozome-next.jgi.doe.gov/ (accessed on 23 December 2019)). Using the comparison of inter-genomic, the mapping of chitinase genes were performed, and TBtools software (https://bio.tools/tbtools (accessed on 24 December 2019)) was used to draw a syntenic diagram. By using the criteria of physical positions of chitinase genes in the genome of cultivated soybean, we identified the tandem duplications. Tandem duplication genes are considered as those that are separated by not more than one intervening gene.

### 2.6. Plant Materials and Culture

To sterilize the seeds of soybean (*W82*), we initial used ethanol (70% *v*/*v*) for 1 min, and after this for 6 min these seeds were bleached (10%); this was followed by sowing them in a 10 cm diameter pot containing vermiculite and nutritive soil at 1:1 (*v*/*v*) mixture. The soybean seedlings were raised in a growth chamber by maintaining the controlled conditions followed by Aleem et al. [25]. After every four days, seedlings were supplied with water in half-length Hoagland solution. The V3 stage of the seedlings were selected for the stress treatments, i.e., fungus inoculation and osmotic stress treatment.

### 2.7. Pathogenic and Drought Treatments

The white mold pathogen of soybean (*Sclerotinia sclerotiorum)* was cultured by following the detailed procedure described by Hoffman et al. [26]. The drop-mycelium method was used for the inoculation of *Sclerotinia sclerotiorum* to soybean leaves, using four replications [27]. The experiments were conducted in controlled conditions at the Soybean Research Institute, Nanjing Agricultural University, China. The *S. sclerotiorum* isolate 105 HT was provided by the Department of Plant Protection, Nanjing Agricultural University and used in disease evaluation. Procedures for the controlled evaluation of white mold diseases in soybean were followed, as described by Chen and Wang, [27]. For about three to four days, potato dextrose agar (PDA) medium was used to grow the sclerotia (sterilized), and fresh stock was maintained by re-culturing the sclerotia. Small pieces of mycelia were put into the liquid broth of potato dextrose, and homogenization of the potato dextrose broth was performed in a G10 Gyrotory shaker (Edison, NJ) at 200 rpm for four nights. A household blender was used to homogenize the suspension of mycelia for maintaining mycelium uniformity immediately before the inoculation. A battery-operated hand sprayer was used to spray a suspension of blended mycelia at ~4.6 ml/plant on the plant leaves, and this spray was used at the V3 growth stage. The inoculated plants were placed in controlled chambers, maintaining near 100% humidity inside the chambers. A control was also used, that was not inoculated with the pathogen.

Seedlings were randomly grouped in four replicates for the osmatic treatments. Three replicates were subjected to drought stress and treated using 20% PEG-6000, whereas the fourth one was used as control, and not subjected to drought treatment. Collection of fresh and healthy leaf tissues was carried out for both control and treated plants (in case of both disease and drought stress) at time intervals of 6, 12, 24 and 48 h post-inoculation (hpi)/post-treatment for the extraction of RNA, and were rapidly flash frozen in liquid nitrogen and stored at −80 °C.

### 2.8. qRT-PCR Analysis

Total RNA was extracted from the leaf tissue (100 mg) that was collected from soybean plants using a PureLink RNA Mini Kit (Ambion Life Technologies, 5791 Van Allen Way Carlsbad, CA, USA). A nanodrop spectrophotometer (Thermo Scientific, Wilmington, DE, USA) was used for checking the quality and quantity of RNA. The protocol used for cDNA synthesis was same as followed by us in the previous study of Sharmin et al. [27]. The primers used in the qRT-PCR analysis are listed in the Table S1. The qRT-PCR reaction was performed as initial annealing at 95 °C for 5 min, followed by 40 cycles as 94 °C for 30 s, 60 °C for 30 s, and 72 °C for 30 s. The reaction mixture and replication used is as per our previous study [28,29].

In our experiment, we used the actin gene as an internal control, and relative expression of each gene was estimated by the Delta Ct method [30]. The *p <* 0.05 was used to check the level of significance.

### 2.9. Statistics

In our experiments we used replicates of three, and every replicate was repeated three times. Student’s *t*-test was used to check for significance differences in gene expression of chitinases. In all experiments, the difference among the groups is reported as statistically significant (* p < 0.05) or extremely significant (** p < 0.01).

## 3. Results

### 3.1. Chitinase Genes Identified in the Glycine max Genome

Soybean whole-genome sequence availability has allowed the characterization of novel gene families in these crop plants, but it requires already known orthologs query genes from the model plants. Therefore, by using the known sequence of 24 chitinase genes of *A. thaliana* as a query, we identified the 38 chitinase genes in cultivated soybean (Table 1). These sequences were further subjected to functional annotation using the Conserved Domain Database (CDD), and the results revealed that the predicted protein sequence of these genes possess either the Glyco_hydro 18 or Glyco_hydro 19 domain (Table 1). These domains are the key component needed by the chitinase enzymes to hydrolyze the chitin; therefore, this confirmed their role as chitinase enzymes. Protein sequences containing the catalytic domain of Glyco_hydro 18 are members of either Class III or V, whereas those possessing Glyco_hydro 19 are the members of any of the three different classes, Class I, II or IV. Interestingly, out of the 38 identified chitinases in soybean, 25 possess Glyco_hydro 18, while only 13 harbored the Glyco_hydro 19 domain (Table 1).

### 3.2. Phylogenetic Analysis and Chromosomal Location of Chitinase in Glycine max 

**Figure 1:**
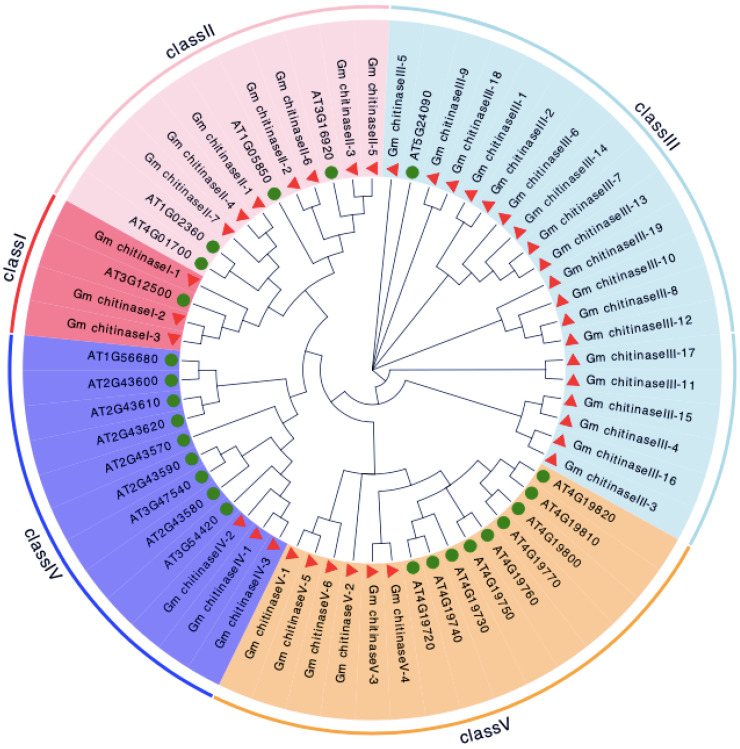
Phylogenetic analysis and chromosomal distribution of chitinase genes identified in the soybean genome.

The protein sequence of the 38 chitinases of soybean, along with the 24 known chitinases from *A. thaliana,* were utilized for developing an unrooted maximum likelihood phylogenetic tree (Figure 1). Based on the phylogenetic relationship, chitinases are classified into five different groups representing five classes of chitinases, I, II, III, IV and V (Figure 1). Each class of chitinase is grouped into separate cluster. Broadly, chitinases are grouped into two mega-groups. All the chitinases of classes I, II and IV, comprising the GH19 family, are clustered into mega-group 1, while mega-group 2 possesses the chitinases of the GH18 family. Naming of chitinases for *Glycine max* is based on their known ortholog of *A. thaliana*, which shows three, seven, nineteen, three and six chitinases of class I, class II, class III, class IV and class V, respectively.

By analyzing the distribution of the chitinase genes on the different chromosomes in soybean, we identified that all of the 38 chitinase genes are distributed on 17 of the total of 20 soybean chromosomes (Figure S1). Distribution of these chitinase genes was random and uneven across the soybean genome. For example, Chr.15 possess four genes, whereas Chr.04, Chr.06 and Chr.14 possess no chitinase gene; however, the remaining chromosomes contain one to three genes. Hence, the results of current study showed that *Glycine max* chitinases are not evenly distributed in the soybean genome.

### 3.3. Structural Analysis of Chitinase Genes in Glycine max

**Figure 2:**
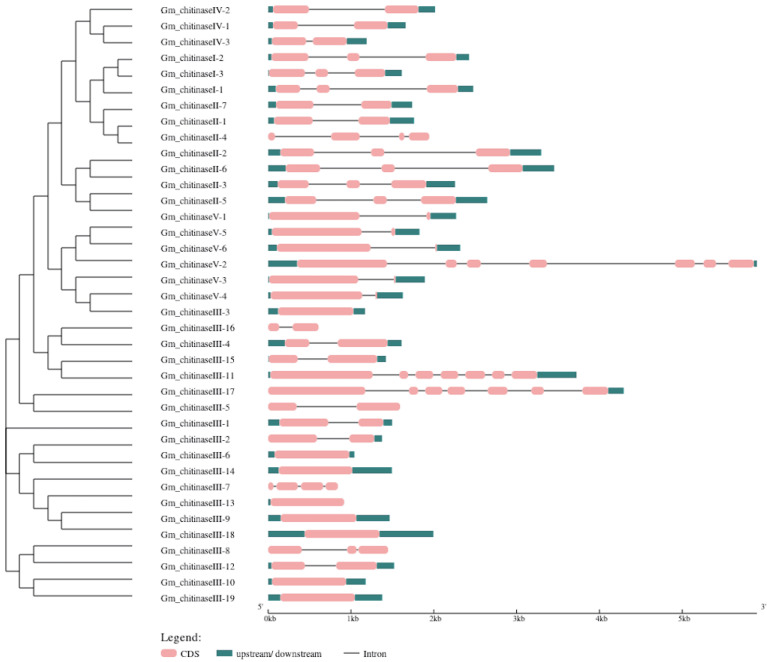
Exon–intron analysis of chitinase genes of soybean. Graphic representation of the gene models of 38 *GmChis* genes identified from *Glycine max*. L genome revealed presence of varied numbers of introns. Exons are shown as red boxes and introns are shown as black lines.

Exon–intron analysis of soybean chitinase genes was carried out by comparing the genomic and coding sequence of each gene (Figure 2). Structural analysis showed that most of the genes of same chitinase class possess almost the same number of exons or introns. For instance, all the three chitinases of class I have two introns; similarly, chitinase genes of class IV and class V contain one intron, except *Gm_chitinaseV-2* of class V, that possesses six introns. Moreover, out of seven chitinases of class II, four have two introns, two have one intron and one has three introns. However, the 19 chitinase genes of class III are very diverse in terms of intron number, which varies from 0–6 introns; for example, eight of them contains zero introns, another eight possess one intron, and the remaining one has three, one has two and two have six introns. Overall, structural analysis revealed that soybean chitinases showed significant variation in exon and intron numbers, and this ultimately leads to differences in the length of different chitinases and their physio-chemical properties (Table 1).

To understand the role and response of the chitinases in plant growth and multiple plant stresses, 1.5 kb upstream promoter sequences of ten randomly selected chitinase genes (two each from classes I, II, III, IV and V) were utilized for cis-regulatory element identification (Figure S2; Table 2). Our results showed the presence of multiple cis-elements regulating the response against biotic and abiotic stresses. For example, biotic stress responsive elements were observed as EIRE (fungal elicitor responsive elements), Box-W, TCA-element (SA-responsive element), CGTCA-motif and TGACG-motif (JA responsive element) and TC-rich repeats (ATTTTC). Similarly, abiotic stress response cis-elements were identified in the chitinase promoter genes such as LTRE motif (TGG/ACC GAC), involved in cold/chilling response, MBS/MYB motif (TAACTG) for water-deficit, HSE motif (CNNGAANNTTCNNG), involved in heat stress, WUN-motif, involved in wound response and ABREs motif (ACGT), regulated by expression of ABA. To this end, many elements showing responsiveness for hormones are also identified, such as gibberellin- (P-box and GARE-motif), ethylene- (ERA) and auxin-responsive elements (TGA) (Table 2). The presence of these elements in the chitinase promoters suggests their regulatory role in multiple abiotic and biotic stresses.

### 3.4. Molecular Modeling of Chitinases in G. max

**Figure 3:**
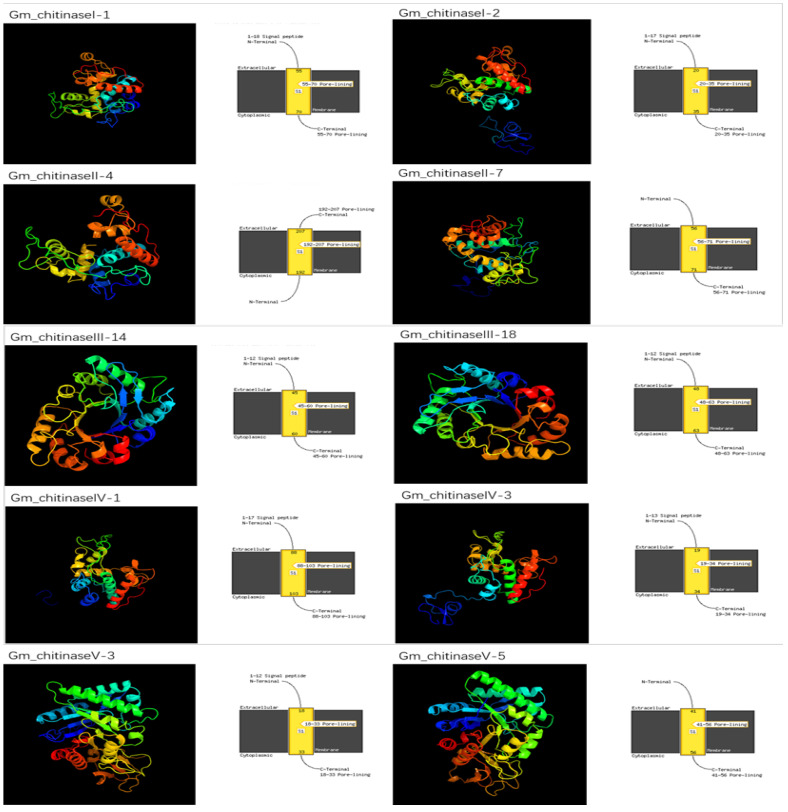
Predicted 3D structures and transmembrane helix (TM) of 10 randomly selected soybean chitinase proteins, two from each class I–V, from top to bottom.

Dynamic and energetic information regarding the chitin binding domain of the chitinase proteins can be determined by using the bioinformatic approach of molecular modeling. This information is very laborious and expensive to obtain, as well as taking a long time. The PHYRE2 server, freely available online, was used to construct 3D models for chitinases of I–V classes, and this analysis provides a better understanding about the structural properties of chitinase genes in soybean (Figure 3). The following parameters were used to generate the 3D model of chitinase proteins: confidence >90% and residue coverage of 72–98. These predicted 3D protein structures can serve as the preliminary basis to understand the function of chitinase genes at the molecular level. Our results revealed that, except class II members, all of the chitinases have a N-terminal signal peptide that possesses a different number of amino acids; however, all the five classes of chitinases possess pore linings with varying amino acid numbers. A signal peptide at the N-terminal guides chitinase proteins to their proper location, and after they reaches their destination, the signal peptide is cleaved off. In addition, results showed the cytoplasmic nature of all chitinases, and extra-cellular mode of action (Figure 3).

### 3.5. Synteny Analysis of Chitinases

**Figure 4:**
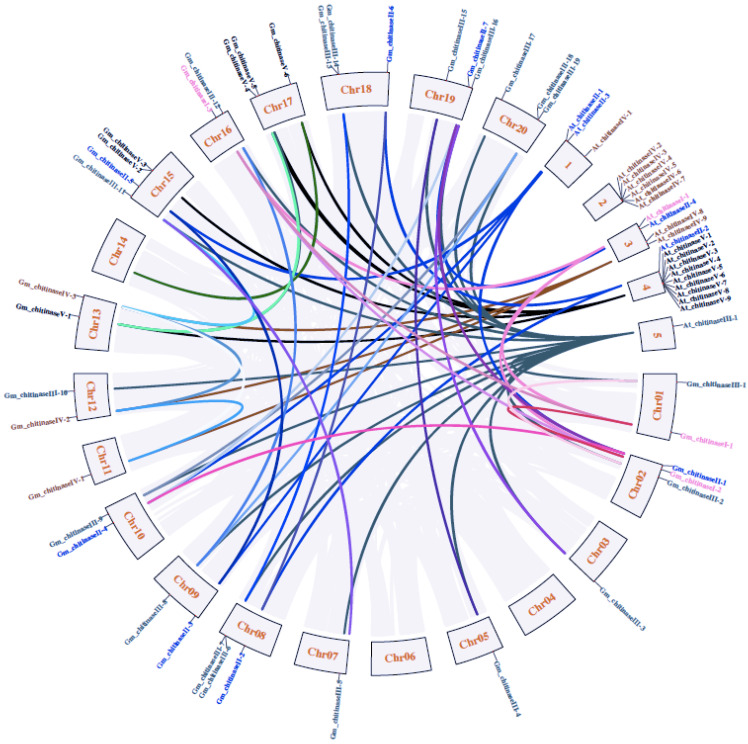
Syntenic relationships of among *A. thaliana and G. max* L. chitinase genes are indicated in different colors. Synteny relationships were lined by Circos (http://circos.ca/ (accessed on 23 December 2019)).

Soybean crops have encountered different duplication events, such as one WGD and WGT events, during their evolution [31]; these events give rise to many copies of different soybean genes, and a highly duplicated genome [32]. Hence, it is expected that each *Arabidopsis thaliana* chitinase gene might have multiple copies in the soybean genome. In this context, we identified only 38 chitinase orthologs from the 24 chitinase genes of *Arabidopsis thaliana*. It is interesting these 38 chitinase genes represent the orthologs of only nine chitinase genes of Arabidopsis, i.e. *At_chitinaseI-1*, *At_chitinaseII-1*, *At_chitinaseII-2*, *At_chitinaseII-3*, *At_chitinaseII-4*, *At_chitinaseIII-1*, *At_chitinaseIV-9*, *At_chitinaseV-7* and *At_chitinaseV-8*. The remaining 15 chitinase genes of *Arabidopsis thaliana* do not have any orthologs in the soybean genome, perhaps because these chitinase genes have been lost during the evolution of the soybean genome. The highest number of 19 ortholog genes was observed for *Arabidopsis* At_chitinaseIII-1 in the soybean genome, followed by three genes each for At_chitinaseI-1, At_chitinaseIV-9, At_chitinaseV-7 and At_chitinaseV-8 and two genes each for At_chitinaseII-1, At_chitinaseII-2, At_chitinaseII-3. At_chitinaseII-4 has the lowest, one ortholog gene, in the soybean genome. The Circos and synteny analysis showed that both tandem duplication and segmental duplication are involved in the expansion of the chitinase gene family in the soybean (Figure 4).

### 3.6. Transcriptional Analysis of Chitinase Genes in Response to White Mold and Drought Stress

**Figure 5:**
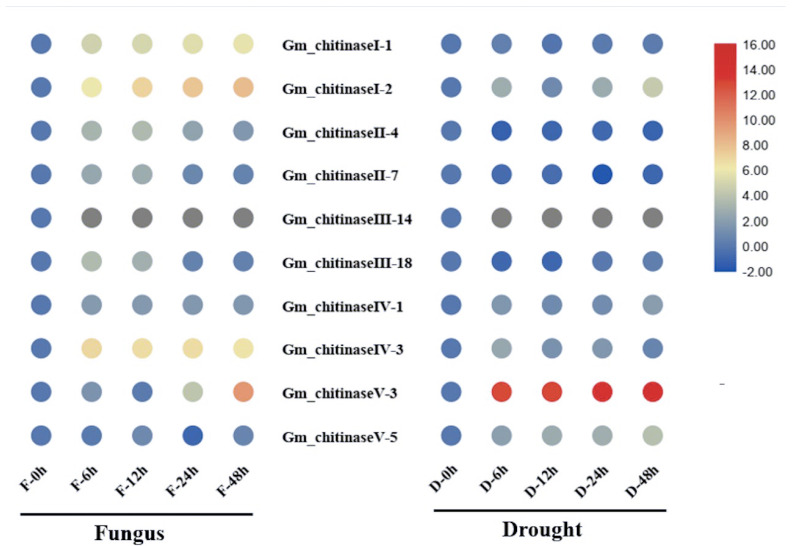
Expression analysis of ten randomly selected chitinase genes (two from each of the five classes) of the soybean ate 6, 12, 24 and 48 h after the inoculation of white mold pathogen (*Sclerotinia sclerotiorum)* and drought stress treatment. Three biological replicates were used to calculate error bars, based on standard error.

Research evidence has revealed the regulatory role of chitinases in biotic stress such as antifungal disease resistance [3,10,33], and abiotic stress such as drought [11,34,35,36]. In addition, the role of chitinases in modulating plant growth and productivity has been also reported [37]. Hence, the current investigation examined changes in the expression of the genes in response to white mold fungal pathogen (*Sclerotinia sclerotiorum*) and drought stress (Figure 5). In this regard, we randomly selected two *GmChis* genes from each of five different classes (I–V) of chitinases identified in the soybean to determine their expression pattern in response to pathogen infection and drought stress. Our results revealed that chitinase of different classes showed a considerably varied response under both pathogen and drought stresses. For example, the chitinases belonging to class I and class III were significantly up-regulated (6-fold to 10-fold) at different intervals following pathogen infection. In contrast, the chitinases of class-II, class IV and class V did not show any significant response under the pathogen treatment. Under drought stress, only the chitinase of class V showed significantly higher up-regulation (up to a 16-fold increase in expression) at all the four time intervals (6 h, 12 h, 24 h and 48 h) following the stress treatment. Chitinases of the remaining four classes did not significantly change under drought stress. This suggests a diverse and specific role of different chitinase genes of soybean in the regulation of biotic and abiotic stresses. Hence, research efforts are needed to functionally elucidate the role of chitinase genes in the regulation of different biotic and abiotic stresses in soybean.

## 4. Discussion

Plants, being immobile, often encounter various environmental stresses, leading to negative effects on the plants’ growth [1]. Plants possess well established defense mechanism to alleviate these stresses. For example, PR proteins are a diverse range of proteins produced by the plants in response to stress, and chitinases are one class of PR proteins that are ubiquitously found in prokaryotes and eukaryotes, including plants [3,38]. Chitinases regulate plant growth and development under biotic (such as fungal pathogens) and abiotic stresses [3,5]. Research investigation has confirmed the important role of chitinases in plant defense, but there is a need to identify and elucidate the function of these genes for their potential use in crop improvement. To date, little is known about chitinases in cultivated soybean (*Glycine max* L.), and no systematic investigation has been carried in soybean. Hence, we undertook a comprehensive and systematic investigation to identify and characterize the chitinase gene family across the whole soybean genome. We identified 38 chitinase genes in the soybean genome, and this number was relatively higher than previously reported in *A. thaliana* [5]. This can be explained as follows: the soybean genome is complex, and in its evolutionary history it has gone through the events of WGD and WGT, ~13–130 million years ago, which might have created multiple gene copies [31]. However, soybean possesses a very similar number of chitinases to what has previously been reported in rice (37), grape (38), *B. rapa* (33) and cucumber (28) [39,40,41,42]. In contrast, soybean possesses a lower number of chitinase genes than *Gossypium hirsutum* (92), *Gossypium barbadense* (116), *E. grandis* (67) and *C. sativa* (79) [3,5,43]. This can be attributed to the large genome size and more duplication events present in these species, compared to soybean [3]. Moreover, chitinase genes in *Glycine max* L. are unevenly and randomly distributed in 17 of the 20 chromosomes (Figure S1). Chen et al. [41] also reported the distribution of 33 chitinases genes on eight of the 10 total chromosomes. Similar findings were observed in rice [39] and *P. trichocarpa* [44].

Based on the phylogenetic relationship, soybean chitinase, along with the known chitinases of *Arabidopsis thaliana,* are classified into five separate clusters, and these five clusters represent five chitinase classes, i.e., I, II, III, IV and V in soybean. From a broader viewpoint, these five clusters are basically separated into two mega-clusters (“mega-cluster 1” & “mega-cluster 2”). The GH19 family chitinases that include class I, II and IV are grouped in “mega-group 1”, and “mega-group 2” possess the chitinases of the GH18 family (class III and V). However, GH19 and GH18 are distinct from each other, as well as having an independent history of evolution [3]. For example, chitinases of the GH18 family possess the catalytic domains triosepho-sphateisomerise (TIM barrel) with highly conserved motif (DxDxE), and these chitinases function in hydrolytic reactions, whereas chitinases of the GH19 family contains alpha-helices and catalyze single displacement [45,46,47]. The chitinase classes of I and II are grouped close to each other, because class II has originated from class I via chitin-binding domain insertion [48]. In addition, the two mega-clusters can be easily identified based on their domain; for example, “mega-cluster 1” chitinases are characterized by the Glyco_hydro_19 domain, whereas “mega-cluster 2” possess the Glyco_hydro_18 domain. Chitinases of “mega-cluster 2” are present in diverse living organisms, such as microorganisms, animals and plants; in contrast, the chitinases of “mega-cluster 1” are uniquely found in plants [44]. However, our results showed that the soybean genome possesses a lower number of GH19 chitinases (13) than GH18 chitinases (25). Similar differences in the contribution of GH18 and GH19 genes to the chitinase family has been also previously reported in *B. rapa* [41], *Musa acuminata* [49] and *Zea mays* [50], etc.

Stress-related genes have been observed to contain a smaller number of introns, relative to other genes that possess no role in plant stress response (Jeffares et al. 2008). Hence, our study showed that, out of 38 chitinase genes identified in the soybean, 36 possess three or fewer introns, and confirmed the above conception. Similar findings were recently reported by Mir et al. [3], who also reported fewer introns in the chitinase genes of *B. juncea* and *C. sativa*. Moreover, many authors have reported lower intron numbers in different stress-related genes such as the LEA family [51], leucine-rich repeat (LRR) family [52] and the trehalose-6-phosphate synthase gene family [53]. Genes that possess a higher number of introns need a longer time for transcription, hence the product of these genes is not available immediately for cellular function. In contrast, genes with reduced intron numbers are quickly transcribed, and are thus rapidly available for defense response [54]. In this context, the reduced number of introns in the soybean chitinase genes allows them to react quickly and respond to stress conditions immediately.

In order to understand chitinase functioning in the various stress responses, we scanned the 1.5 kb upstream promoter regions of chitinase genes for cis-elements. The bioinformatic analysis revealed the presence of multiple cis-regulatory elements, either in one or more copies, in the upstream promoter regions. The biotic stress regulatory cis-elements present in the promoter region include SA motifs, TC-rich repeats, JA motifs and fungal responsive elements. Hence, this suggests a function of chitinase in modulating the stress response in plants. The ABA-dependent pathway activates the genes involved in the abiotic stress response in plants, and it requires the presence of single or multiple copies of ABREs motifs. In addition, these genes are activated independently via binding of different DREBPs groups to DRE motifs (TAC CGA CAT) [55]. To this end, the MYB and MBS cis-elements identified in the upstream region are drought-inducibility elements/motifs, suggesting role of the chitinase in drought stress [56]. Additionally, cold/chilling responsive cis-elements (LTRE) were also identified [57], and HSEs are the important cis-elements present in the heat shock protein genes (HSPs) regulating the heat stress response in plants [58]. Moreover, the presence of ERA, GARE- motif, P-box and TGA-element in the chitinase promoters suggests their regulatory influence by plant hormones. The motifs of SA and JA are present in many stress-related genes and regulate stress tolerance in plants [4]. Similar to our findings, these motifs (existing in one or more copies) were also previously reported in chitinase genes and other PR genes in different plants, such as *B. juncea* and *C. sativa* [3,4], and thus our results provide preliminary evidence for the functioning of chitinase genes in multiple plant stresses. Therefore, cis-regulatory element analysis showed that soybean chitinase might be involved in modulating both biotic and abiotic stress tolerance in soybean.

Widening of gene families occurs through different types of duplication events, such as WGD/WGT, segmental and tandem duplications [59]. The two and one WGD and WGT events experienced by soybean genome in its evolution have produced many copies of soybean genes and led to the genome’s complexity [31,32]. However, all *A. thaliana* chitinase genes do not have homologous genes in the soybean genome; only nine chitinase genes of *Arabidopsis* possess homologs in the soybean genome. The remaining 15 chitinase genes of *Arabidopsis* do not have any orthologs in the soybean genome, perhaps because these chitinase genes have been lost during the evolution of the soybean genome. Interestingly, *At_chitinaseIII-1* has 19 chitinase orthologs in the soybean genome, and they represent mostly tandem duplications, but a few are segmental duplications. Four genes, *At_chitinaseI-1*, *At_chitinaseIV-9*, *At_chitinaseV-7* and *At_chitinaseV-8,* revealed triplication, and this has evolved through tandem duplication. The remaining three genes, *At_chitinaseII-1*, *At_chitinaseII-2* and *At_chitinaseII-3,* showed duplication, and this has also evolved through tandem duplications, and *At_chitinaseII-4* has only a single copy in the soybean genome. Hence, the widening of the soybean chitinase gene family has mainly resulted from tandem duplications (Figure 4). Our results suggest that *Arabidopsis* chitinase genes might have been conserved before speciation, but have been lost during the evolution of the soybean genome as well as during artificial selection. Similar to our findings, the homologs of *Arabidopsis* chitinase has been lost in other plant species as well. For example, 10 *Arabidopsis* chitinase genes do not have orthologs, and are lost in *B. rapa* [41]. Similar findings were observed in *B. Juncea* and *C. sativa* by Mir et al. [3]. In addition, the WGD and WGT events leading to the loss of genes in soybean have been reported for other gene families, such as cytokinin oxidase/dehydrogenase (CKX) genes [60], nucleotide binding site (NBS)-encoding genes [61] and MKK and MPK genes [62]. These results suggest that expansion or elimination of some *Arabidopsis* chitinase genes in the soybean genome might have occurred due to functional differentiation of these genes under diverse environmental stresses. The soybean probably has retained a sufficient number of chitinase genes during its evolution to respond to external stress properly. 

In plants, PR proteins modulate the plant defense system to provide protection against various environmental stresses. Hence, the PR-3 family of PR proteins represents the chitinases class [9], and expression of PR-3 proteins has been demonstrated to be induced by both biotic and abiotic stresses [5,11]. Therefore, our results revealed that chitinases belonging to specific classes were significantly induced under white mold fungal pathogen and drought stress treatments. For example, the chitinases belonging to class I and class III were significantly up-regulated (6-fold to 10-fold) at different intervals following pathogen infection. In contrast, the chitinases of class II, class IV and class V did not show any significant response under the pathogen treatment, which is similar to reports of different studies in various plants [63,64,65]. Moreover, in the cotton plant, the expression of chitinase genes was induced by inoculation of a pathogen (*Verticillium dahlia*) and significantly reached peak level 24 h following inoculation [5]. Under drought stress, only the chitinases of class V showed significantly higher up-regulation (up to a 16-fold increase in expression) at all the four time intervals (6 h, 12 h, 24 h and 48 h) following the stress treatment. Chitinases of the remaining four classes did not undergo significant changes under drought stress. In agreement with our report, chitinase expression induced by drought stress has also been reported in *Arabidopsis thaliana* [11] and *Crocus sativus* [20]. Hence, the above findings suggest an important role of chitinase genes in controlling multiple plant stress (diseases and abiotic) responses in soybean plants. Therefore, the more research efforts are required to elucidate the detailed function and mechanism involved in chitinase-mediated regulation of plant defense.

## 5. Conclusions

The current investigation provides a comprehensive and systematic report of the chitinase gene family at the whole genome scale in soybean. Here, we detected 38 chitinase genes in the soybean genome, and these genes were randomly and unevenly distributed on the soybean chromosomes. Phylogenetic analysis grouped these chitinase genes into five distinct clusters representing five classes of chitinase (I, II, III, IV and V). In addition, synteny and duplication analysis revealed that tandem duplication has played the major role in widening the family of chitinase genes in soybean, while segmental duplication has the smallest role. Promoter analysis showed multiple cis-regulatory elements related to biotic and abiotic stresses in the upstream region of the chitinase genes, suggesting their role in plant defense response against multiple stresses. Moreover, gene expression analysis revealed that pathogenic and drought stress treatments significantly induce the up-regulation of chitinase genes belonging to specific classes at different time intervals, which further confirmed their role in plant stress response. Overall, our study provides evidence about the role of the chitinases in multiple plant stress responses in soybean. However, there is a need for future research efforts to validate the specific or general functions of different chitinases against different biotic and abiotic stresses. Therefore, extensive research efforts are required to elucidate the detailed mechanism involved in chitinase-mediated modulation for different plant stresses, for their potential use in soybean improvement.

## Figures and Tables

**Table 1:** Genome-wide identification and distribution of chitinase genes in soybean.

S. No.	Name	Gene ID	Class	Protein Length(aa)	Mol. Wt.(Da)	PI(pH)	Instability Index	GRAVY	*Arabidopsis* Ortholog Locus	*Arabidopsis* Locus Description
1	Gm_chitinaseI-1	Glyma.01G160100	Ⅰ	275	30,182.55	5.34	38.39	−0.331	AT3G12500	BASIC CHITINASE, PR3
2	Gm_chitinaseI-2	Glyma.02G042500	Ⅰ	320	34,341.3	7.40	29.21	−0.404	AT3G12500	BASIC CHITINASE, PR3
3	Gm_chitinaseI-3	Glyma.16G119200	Ⅰ	317	34,445.61	8.10	38.56	−0.350	AT3G12500	BASIC CHITINASE, PR3
4	Gm_chitinaseII-1	Glyma.02G007400	Ⅱ	281	31,229.36	8.83	49.95	−0.290	AT1G02360	Chitinase family protein
5	Gm_chitinaseII-2	Glyma.08G259200	Ⅱ	326	36,029.92	5.83	34.73	−0.180	AT1G05850	CHITINASE-LIKE protein 1
6	Gm_chitinaseII-3	Glyma.09G038500	Ⅱ	317	34,709.20	7.01	34.06	−0.266	AT3G16920	Encodes a chitinase-like protein
7	Gm_chitinaseII-4	Glyma.10G138400	Ⅱ	245	27,411.72	8.66	42.22	−0.578	AT1G02360	Chitinase family protein
8	Gm_chitinaseII-5	Glyma.15G143600	Ⅱ	318	34,889.50	6.97	34.32	−0.254	AT3G16920	Encodes a chitinase-like protein
9	Gm_chitinaseII-6	Glyma.18G283400	Ⅱ	329	36,375.35	5.91	37.68	−0.176	AT1G05850	CHITINASE-LIKE protein 1
10	Gm_chitinaseII-7	Glyma.19G221800	Ⅱ	272	29,960.98	6.80	36.74	−0.181	AT4G01700	Chitinase family protein
11	Gm_chitinaseIII-1	Glyma.01G055200	Ⅲ	296	31,735.72	5.39	35.54	−0.113	AT5G24090	Chitinase A (class III)
12	Gm_chitinaseIII-2	Glyma.02G113600	Ⅲ	296	31,687.55	5.18	34.54	−0.106	AT5G24090	Chitinase A (class III)
13	Gm_chitinaseIII-3	Glyma.03G254300	Ⅲ	303	32,588.90	8.97	38.48	−0.231	AT5G24090	Chitinase A (class III)
14	Gm_chitinaseIII-4	Glyma.05G075000	Ⅲ	298	32,643.35	9.41	38.72	−0.115	AT5G24090	Chitinase A (class III)
15	Gm_chitinaseIII-5	Glyma.07G061600	Ⅲ	289	31,297.28	6.31	23.17	−0.068	AT5G24090	Chitinase A (class III)
16	Gm_chitinaseIII-6	Glyma.08G299700	Ⅲ	300	32,004.46	8.08	38.61	0.024	AT5G24090	Chitinase A (class III)
17	Gm_chitinaseIII-7	Glyma.08G300300	Ⅲ	245	25,864.39	4.87	35.41	0.106	AT5G24090	Chitinase A (class III)
18	Gm_chitinaseIII-8	Glyma.09G126200	Ⅲ	292	30,880.19	4.07	33.02	−0.016	AT5G24090	Chitinase A (class III)
19	Gm_chitinaseIII-9	Glyma.10G227700	Ⅲ	304	32,429.85	7.58	39.30	0.049	AT5G24090	Chitinase A (class III)
20	Gm_chitinaseIII-10	Glyma.12G156600	Ⅲ	298	31,508.36	5.51	30.94	−0.050	AT5G24090	Chitinase A (class III)
21	Gm_chitinaseIII-11	Glyma.15G015100	Ⅲ	820	91,012.67	6.31	35.59	−0.141	AT5G24090	Chitinase A (class III)
22	Gm_chitinaseIII-12	Glyma.16G173000	Ⅲ	297	31,768.55	5.01	34.00	−0.043	AT5G24090	Chitinase A (class III)
23	Gm_chitinaseIII-13	Glyma.18G120200	Ⅲ	295	31,225.32	5.87	35.97	0.095	AT5G24090	Chitinase A (class III)
24	Gm_chitinaseIII-14	Glyma.18G120700	Ⅲ	295	31,266.46	7.50	32.96	0.045	AT5G24090	Chitinase A (class III)
25	Gm_chitinaseIII-15	Glyma.19G076200	Ⅲ	316	34,753.54	9.42	37.34	−0.238	AT5G24090	Chitinase A (class III)
26	Gm_chitinaseIII-16	Glyma.19G251900	Ⅲ	148	16,384.63	8.91	37.51	−0.124	AT5G24090	Chitinase A (class III)
27	Gm_chitinaseIII-17	Glyma.20G035400	Ⅲ	800	88,944.66	7.93	42.27	−0.170	AT5G24090	Chitinase A (class III)
28	Gm_chitinaseIII-18	Glyma.20G164600	Ⅲ	301	32,393.00	9.34	41.78	−0.017	AT5G24090	Chitinase A (class III)
29	Gm_chitinaseIII-19	Glyma.20G164900	Ⅲ	299	32,114.60	4.27	38.89	−0.092	AT5G24090	Chitinase A (class III)
30	Gm_chitinaseIV-1	Glyma.11G124500	Ⅳ	235	25,871.79	4.90	34.87	−0.261	AT3G54420	CHITINASE CLASS IV
31	Gm_chitinaseIV-2	Glyma.12G049200	Ⅳ	280	30,569.11	4.94	26.76	−0.276	AT3G54420	CHITINASE CLASS IV
32	Gm_chitinaseIV-3	Glyma.13G346700	Ⅳ	274	29,829.05	5.02	28.58	−0.301	AT3G54420	CHITINASE CLASS IV
33	Gm_chitinaseV-1	Glyma.13G155800	Ⅴ	379	41,065.40	4.78	16.18	0.141	AT4G19800	Glycoside hydrolase, family 18
34	Gm_chitinaseV-2	Glyma.15G206400	Ⅴ	762	86,075.18	6.40	39.94	−0.167	AT4G19800	Glycoside hydrolase, family 18
35	Gm_chitinaseV-3	Glyma.15G206800	Ⅴ	365	40,085.06	8.92	34.77	−0.304	AT4G19810	CLASS V CHITINASE
36	Gm_chitinaseV-4	Glyma.17G076100	Ⅴ	374	41,252.20	8.79	33.08	−0.102	AT4G19810	CLASS V CHITINASE
37	Gm_chitinaseV-5	Glyma.17G103500	Ⅴ	377	41,059.92	9.11	18.08	0.158	AT4G19800	CLASS V CHITINASE
38	Gm_chitinaseV-6	Glyma.17G217000	Ⅴ	384	43,291.89	6.14	32.33	−0.228	AT4G19810	CLASS V CHITINASE

**Table 2:** Putative cis-regulatory elements in BjPR1 promoter sequence, identified by PlantCARE and PLACE promoter databases.

Cis-Acting Element	Function	Sequence
ABRE	ABA-dependent expression	ACGTG/AACCCGG
ABRE3a	ABA-dependent expression	TACGTG
ABRE4	ABA-dependent expression	CACGTA/CACGTA
AuxRE	part of an auxin-responsive element	TGTCTCAATAAG
CGTCA-motif	JA responsive element	CGTCA
GARE-motif	gibberellin-responsive element	TCTGTTG
GT1-motif	pathogen and salt response	GGTTAA/GTGTGTGAA
LTR	cis-acting element involved in low-temperature responsiveness	CCGAAA
MBS	drought stress	CAACTG
MYB	drought stress	CAACCA/CAACAG/TAACCA
MYB-like sequence	drought stress	TAACCA
MYC	early response to drought and ABA induction	CAATTG/CATGTG/CATTTG
P-box	gibberellin-responsive element	CCTTTTG
TATC-box	cis-acting element involved in gibberellin-responsiveness	TATCCCA
TCA-element	cis-acting element involved in salicylic acid responsiveness	CCATCTTTTT/TCAGAAGAGG
TC-rich repeats	cis-acting element involved in defense and stress responsiveness	ATTCTCTAAC
TGACG-motif	cis-acting regulatory element involved in MeJA-responsiveness	TGACG
TGA-element	auxin-responsive element	AACGAC
W-box	activation of defense and wounding-related genes	TTGACC
WUN-motif	wound response	AAATTACT/TTATTACAT

## Data Availability

Data is contained within the article and Supplementary Materials.

## Supplementary Materials

The following supporting information can be downloaded at: https://www.mdpi.com/article/10.3390/life12091340/s1, Figure S1: Diagram showing the distribution of the 38 chitinase genes identified among the different 17 of the total 20 chromosomes of the soybean; Figure S2: In silico analysis of Chitinase gene promoters of *G. max* L. Promoter cis-elements of 10 chitinase genes (two genes from each five classes of chitinases identified in soybean) in response to biotic, abiotic and hormonal stresses are shown in different shapes and colors along with their respective positions from the start codon ATG; Table S1: List of primers used in the qRT-PCR analysis of the selected chitinase genes of soybean.
