# Supplementary material for: Genome-Wide Identification and Expression Analyses of the Chitinase Gene Family in Response to White Mold and Drought Stress in Soybean (Glycine max)
Source: Life (Basel). 2022 Aug 29;12(9):1340. doi: 10.3390/life12091340 (PMC9504482; doi:10.3390/life12091340)
Supplement: Supplementary file 1 [file life-12-01340-s001.zip › life-1872358-supplementary/Supplemmentary Figures.pdf]

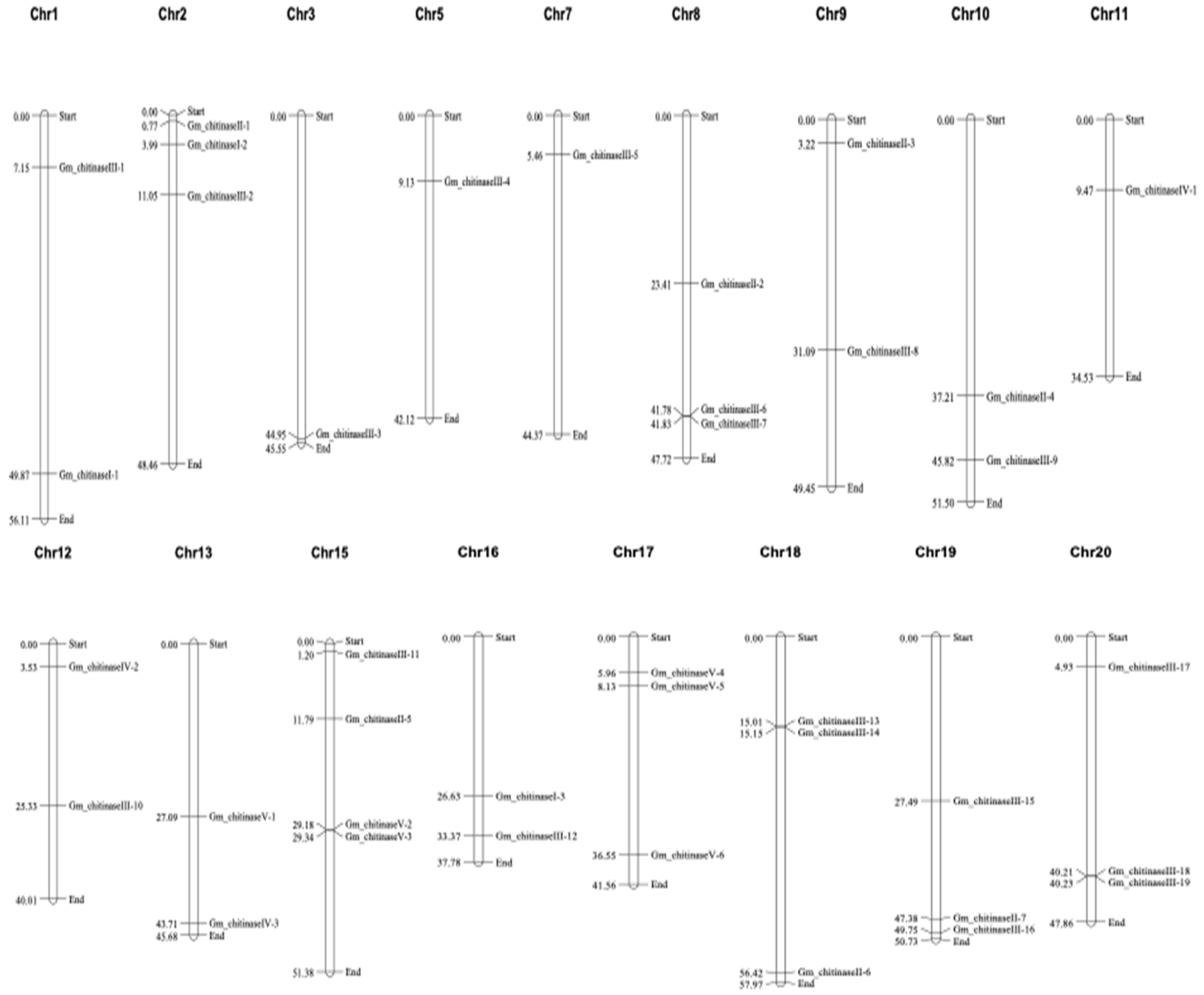

Figure S1: Diagram showing the distribution of the 38 chitinase genes identified among the different 17 of the total 20 chromosomes of the soybean.

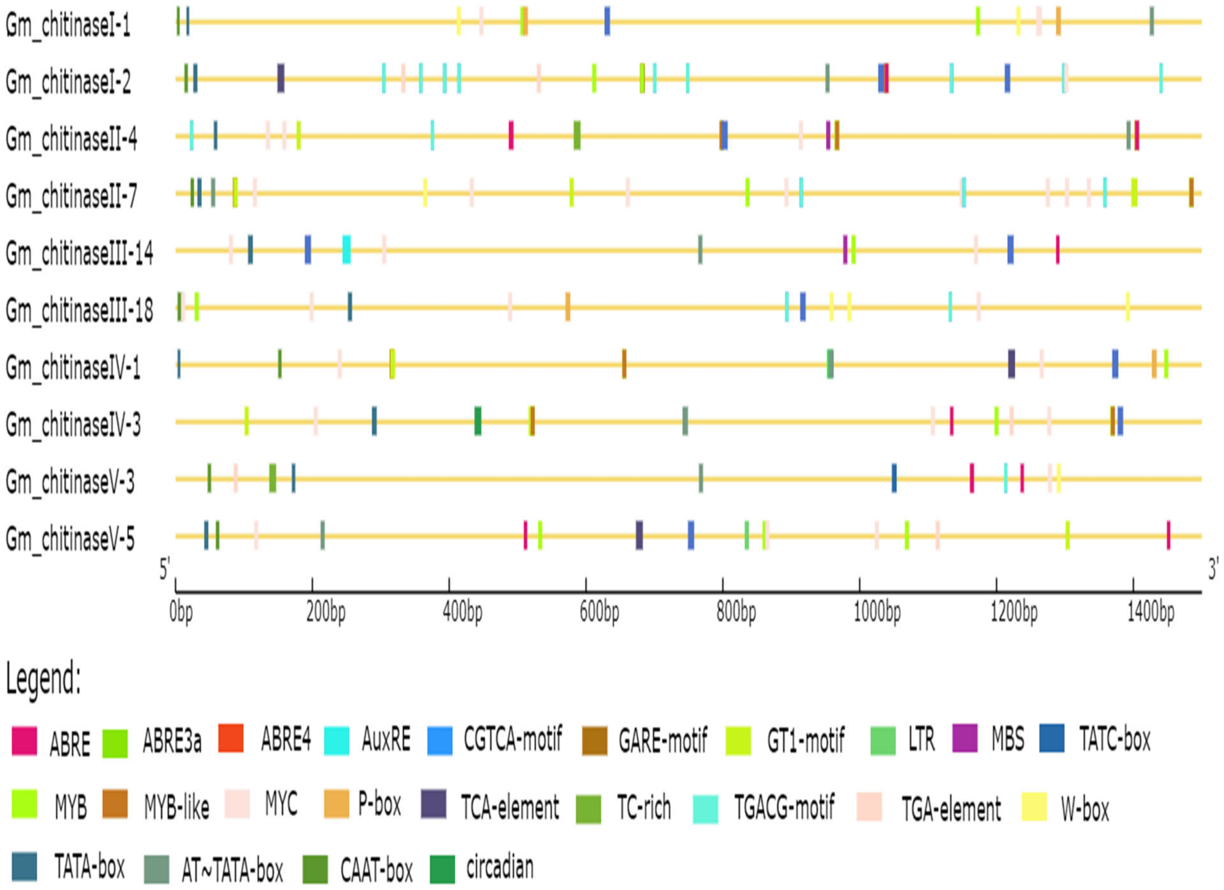

Figure S2: *In silico* analysis of Chitinase gene promoters of *G. max* L. Promoter *cis*-elements of 10 chitinase genes (two genes from each five classes of chitinases identified in soybean) in response to biotic, abiotic and hormonal stresses are shown in different shapes and colors along with their respective positions from the start codon ATG.
