# Supplementary material for: Genome-Wide Identification and Expression Analyses of the Chitinase Gene Family in Response to White Mold and Drought Stress in Soybean (Glycine max)
Source: Life (Basel). 2022 Aug 29;12(9):1340. doi: 10.3390/life12091340 (PMC9504482; doi:10.3390/life12091340)
Supplement: Supplementary file 1 [file life-12-01340-s001.zip › life-1872358-supplementary/Table S1.pdf]

Table S1: List of primers used in the qRT-PCR analysis of the selected chitinase genes of soybean

GeneID	Chitinase Name	Fprimer	Rprimer
Glyma.01g160100	Gm_chitinaseI-1	ACACTATCTTCGCCTTTATCGT	CCCCTCACAAATTTGATCGTTT
Glyma.02g042500	Gm_chitinaseI-2	CAGGATAATCGGGTCCAAGATC	GTTACAATCCAAGTTGTCTCCG
Glyma.10g138400	Gm_chitinaseII-4	GACAAAGTTACAAAGGCAGAGG	GGCACATATTTTCCAACCATCA
Glyma.19g221800	Gm_chitinaseII-7	GCTTTGCTCTATTGCTCTTCTC	CATGCAGTGTCATCTTTGTGAA
Glyma.18g120700	Gm_chitinaseIII-14	TGTCATGGACTTCTAACCTTGC	ACATTCTCGGTAACAAACAGGA
Glyma.20g164600	Gm_chitinaseIII-18	TTGTAAGTTCTTGGAAGCGTTG	CCGTTCTGGACATCAAAGAATC
Glyma.11g124500	Gm_chitinaseIV-1	TACTGTTCAGGCTCGTGTTAAT	TGGATGCACAATATCGATGGTA
Glyma.13g346700	Gm_chitinaseIV-3	GGCAAGAACTTTTACTCACGAG	CATGTTAGTTGAATCGGACCAC
Glyma.15g206800	Gm_chitinaseV-3	GAAAAGACGTAGCCAACAACTT	CGGTGCAAAGAGTCCATTTTTA
Glyma.17g103500	Gm_chitinaseV-5	GCAATGCCTTCATTCAGCATAT	GACTGTTACGCAAAAGGATGAG
